# Spectral and spatial selectivity of luminance vision in reef fish

**DOI:** 10.3389/fncir.2014.00118

**Published:** 2014-09-30

**Authors:** Ulrike E. Siebeck, Guy Michael Wallis, Lenore Litherland, Olga Ganeshina, Misha Vorobyev

**Affiliations:** ^1^School of Biomedical Sciences, The University of QueenslandBrisbane, QLD, Australia; ^2^Centre for Sensorimotor Neuroscience, School of Human Movement Studies, The University of QueenslandBrisbane, QLD, Australia; ^3^Department of Optometry and Visual Science, Auckland UniversityAuckland, AU, New Zealand

**Keywords:** reef fish, operant conditioning, behavior, visual modeling, luminance vision

## Abstract

Luminance vision has high spatial resolution and is used for form vision and texture discrimination. In humans, birds and bees luminance channel is spectrally selective—it depends on the signals of the long-wavelength sensitive photoreceptors (bees) or on the sum of long- and middle-wavelength sensitive cones (humans), but not on the signal of the short-wavelength sensitive (blue) photoreceptors. The reasons of such selectivity are not fully understood. The aim of this study is to reveal the inputs of cone signals to high resolution luminance vision in reef fish. Sixteen freshly caught damselfish, *Pomacentrus amboinensis*, were trained to discriminate stimuli differing either in their color or in their fine patterns (stripes vs. cheques). Three colors (“bright green”, “dark green” and “blue”) were used to create two sets of color and two sets of pattern stimuli. The “bright green” and “dark green” were similar in their chromatic properties for fish, but differed in their lightness; the “dark green” differed from “blue” in the signal for the blue cone, but yielded similar signals in the long-wavelength and middle-wavelength cones. Fish easily learned to discriminate “bright green” from “dark green” and “dark green” from “blue” stimuli. Fish also could discriminate the fine patterns created from “dark green” and “bright green”. However, fish failed to discriminate fine patterns created from “blue” and “dark green” colors, i.e., the colors that provided contrast for the blue-sensitive photoreceptor, but not for the long-wavelength sensitive one. High resolution luminance vision in damselfish, *Pomacentrus amboinensis*, does not have input from the blue-sensitive cone, which may indicate that the spectral selectivity of luminance channel is a general feature of visual processing in both aquatic and terrestrial animals.

## Introduction

Reef fish are famously colorful to human eyes, and often their colors are arranged in complex patterns that vary between species and frequently also between individuals of the same species. Most interest has been directed at understanding the function of these colors for intra- and inter-specific signaling (e.g., Frisch, 1912; Lorenz, 1962; Marshall, 2000; Cheney et al., 2009; Siebeck et al., 2010; Millar and Hendry, 2012) while investigations into visual processing of colors and patterns in fish are still comparatively rare as this field has only started to develop relatively recently. What we have learned about visual processing in fish is often surprisingly similar to what we know about visual processing in primates. Fish extract color information via color opponent cells (Kamermans et al., 1991; Patterson et al., 2002; Ramsden et al., 2008) and they possess direction/orientation selective ganglion cells in the retina which facilitate shape discrimination and the perception of illusory contours (Wyzisk and Neumeyer, 2007; Tsvilling et al., 2012).

Both, fish and primates have typical vertebrate eyes but differ in some aspects of their design, e.g., optics, which is mostly due to differences in their terrestrial/aquatic lifestyles (Land, 1990). Both have a duplex retina with rods and cones, however the spectral sensitivities and number of their photoreceptors differ (Lythgoe, 1979). In addition to single cones, fish also have double cones, which are two photoreceptor cells, which are fused together (Marchiafava, 1985). The function of double cones has long thought to involve motion detection and it was thought that they did not contribute to color vision due to electrical coupling of their two members (Boehlert, 1978). However, a recent study on the trigger fish, *Rhinecanthus aculeatus* showed that in this species, both members do contribute separately to color vision (Pignatelli et al., 2010). The spectral sensitivities and/or color vision abilities of fish have been investigated using a variety of methods, including behavioral experiments (e.g., Neumeyer, 1984; Risner et al., 2006; Siebeck et al., 2008), electrophysiological experiments (electroretinogram or ERG, e.g., Morita et al., 1997; Hughes et al., 1998; Hawryshyn et al., 2010), and microspectrophotometric (MSP) measurements of individual photoreceptor sensitivities (e.g., Losey et al., 2003; Waller, 2005; Marshall et al., 2006). Overall, results show that teleost fish can have up to five photoreceptor sensitivities, but also that not all of them necessarily contribute to color vision simultaneously (Sabbah et al., 2010). The number of different spectral photoreceptor types can therefore not be used to infer the dimensionality of the color vision system.

Color vision requires that the output of at least two photoreceptor types is compared, which is best demonstrated using behavioral experiments (Kelber et al., 2003; Kelber and Osorio, 2010) but can also be shown using ERG recordings under various illumination and background conditions (Hughes et al., 1998). Natural colors differ in hue as well as in brightness, and experiments designed to test color vision therefore must control for luminance cues (e.g., Kelber et al., 2003; Siebeck et al., 2008). This can be done through “gray card experiments” where animals are trained to pick out the colored stimulus from a range of stimuli that differ in brightness (Frisch, 1913). Alternatively, visual modeling can be used to design isoluminant stimuli, which are only discriminable if the animal has color vision, provided the photoreceptor sensitivities are known for the animal under investigation (Vorobyev et al., 2001; Pignatelli et al., 2010).

In a previous study, we showed with behavioral experiments that *Pomacentrus amboinensis* have color vision (Siebeck et al., 2008). The fish were not only able to discriminate yellow from blue of varying brightness levels but they could also generalize from one blue or yellow to other blue or yellow stimuli. We also know that this species is sensitive to ultraviolet (UV) light and uses complex UV patterns to discriminate between conspecific and heterospecific fish (Siebeck, 2004; Siebeck et al., 2010). Microspectrophotometric studies have shown that* P. amboinensis* have four spectral types of cone visual pigments peaking at 365 nm (UV sensitive), 485 nm (short-wavelength sensitive, S), 504 nm (middle-wavelength sensitive, M) and 526 nm (long-wavelength sensitive, L). The UV and middle-wavelength sensitive visual pigments are housed in single cones, while the short-wavelength sensitive visual pigment and long-wavelength sensitive pigments are housed in double cones (Waller, 2005; Siebeck and Hart unpublished results).

In primates, parallel pathways exist for luminance and color processing, which not only differ in their spectral but also spatial properties (Livingstone and Hubel, 1988). In primates the combined outputs of the long-wavelength (L) and middle wavelength (M) sensitive cones contribute to luminance vision, while all three cones contribute to color vision. The luminance channel has high spatial acuity while the color channel has low spatial acuity (Cavanagh et al., 1987). Similar parallel processing of color and luminance has been found in other terrestrial animals (for review see Osorio and Vorobyev, 2005). In honeybees, S, M and L cones contribute to color vision while only the L cones are involved in luminance vision (Backhaus, 1991; Giurfa et al., 1997). In birds, double cones housing the L visual pigment (the spectral sensitivity of the double cones is similar to the sum of L and M cones in primates) contribute to luminance vision and single cones to color vision (Osorio et al., 1999). Overall, it appears that the L-cone is generally involved in luminance vision in terrestrial animals. First hints about a potential similar mechanism in fish came from a study, which found that different spectral sensitivity functions were found when goldfish were trained to discriminate a dark from a light field compared to when the fish were trained to discriminate a light from a dark field (Neumeyer et al., 1991). The authors hypothesized that the when fish were trained on the dark field they learned to discriminate the stimuli based on “color” cues whereas the fish trained on the light field were using “luminance” cues, and proposed that separate color and luminance channels exist in these fish.

The aim of this study was therefore to directly test for the existence of such a luminance channel based on L/M cones and to assess whether this channel has spatial properties similar to the luminance channel found in primates. Specifically, we tested whether *P. amboinensis* are able to discriminate between stimuli with either low spatial frequency (solid colors) or high spatial frequency (checked color patterns) which were designed to be isoluminant for the L-cones and M cones. Visual modeling based on quantum catch calculations was used in order to select specific colors that selectively eliminated the contribution of the L-cones (Vorobyev and Osorio, 1998). Investigating the contribution of the L/M system to high spatial vision is particularly interesting in this species as they are able to discriminate between complex UV patterns when contrast is given in the UV only and fail to discriminate between size matched conspecifics and heterospecifics in the absence of UV signals (Siebeck et al., 2010).

## Materials and methods

### Fish

Fish were collected with hand nets while on SCUBA around Lizard Island, Australia (fisheries permit: PRM37727I; GBRMPA permit G05/13668.1). Throughout experimentation, the fish were maintained in individual aquaria (30 cm × 40 cm × 30 cm) exposed to natural sunlight, given a PVC tube for shelter and supplied with fresh seawater (flow-through system). Aquaria were cleaned daily and fish were fed as part of the experiments. Following the experiments, all fish were released onto the reef where they had been caught. Experiments were conducted during two field trips using 16 (exp 1) and 12 fish (exp 2). All experiments were conducted according to the animal welfare act Australia and approved by the ethics committee of the University of Queensland (ethics permit VTHRC/194/08/ARC/UQ).

### Stimuli

#### General

**Figure 1:**
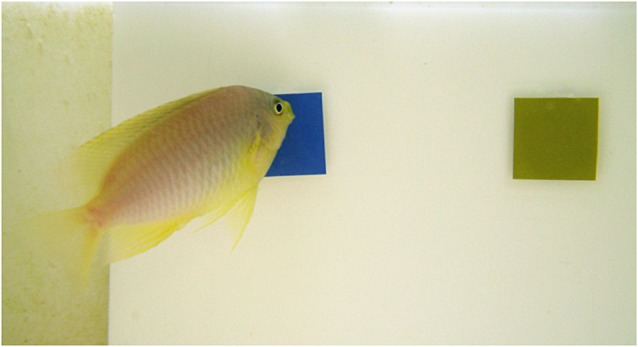
**A *Pomacentrus amboinensis* individual during experiment 1**. The fish indicates its choice by pushing a stimulus with its mouth. Image credit: U.E. Siebeck.

Four sets of stimuli were created by printing (Epson Stylus Photo 1290) the selected colors in patches of 2 × 2 cm on photo paper (Epson glossy photopaper). The squares were then cut out and laminated (Ibico pouchMaster 9VT). Six replicate stimuli were created for each stimulus condition. Three colors were created, a light green, a dark green and a blue (for details see below). The two greens differed in brightness but not in chromaticity, while the dark green and blue were closely matched in terms of their L and M cones quantum catches (Figure 1).

The colors were either combined to patterns (stripes or checkerboards) or left as solid colors to create four conditions: (1) blue/dark-green checkerboards vs. blue/dark-green stripes; (2) dark-green/light-green checkerboards vs. dark-green/light-green stripes; (3) solid blue vs. solid dark-green; and (4) solid dark-green vs. solid light-green (Figure 1).

#### Visual modeling

The spectral reflectance of the stimuli was also measured using the fiber-optic spectrometer and PX-2 pulsed xenon light source. The angle between illumination and measurement probes was held at 45° with a custom made holder fitted with collimating lenses. The receptor quantum catches relative to 100% reflecting white were calculated for each stimulus color. A tetrachromatic visual system was assumed on the basis of the photoreceptor sensitivity data (two single cones (λ_max_ = 365 nm and 504 nm) and one double cone (λ_max_ = 480 nm and 524 nm); S, M, L: Waller, 2005; UV, S, M, L: Siebeck and Hart unpublished results). The receptor spectral sensitivities were calculated using Govardovskii templates (Govardovskii et al., 2000) combined with the ocular media transmittance (Siebeck and Marshall, 2001, 2007). The illumination of the experimental arena was natural daylight with the UV part of the spectrum removed by the material shading the outdoor aquaria. Here, we report the quantum catches calculated using D65 standard daylight spectrum.

The process of identifying the required stimuli involved printing a large series of potential stimuli, laminating them, measuring their reflectance and calculating the quantum catches. This process was repeated until stimuli were found that fulfilled our prerequisites. The spectra of two stimuli with identical chromaticity but different lightness (dark green and light green) were adjusted so that the ratio of quantum catches for all four receptors was constant. The spectrum of a third color (blue) was adjusted so that the L and M-cone quantum catches closely matched the L and M quantum catches of dark green stimulus. (Figure 1).

### Training

The fish were trained using the method described in Siebeck et al. (2009). Briefly, the fish were trained to associate food with a colored stimulus (laminated printout presented on a board inserted into the aquarium for each trial), which they had to “tap” (push with their mouth) in order to receive a food reward (Figure 1). The food delivery was separated from the stimuli in time and location so that no olfactory cues were present while the fish were making their choices. Only once the fish had made a correct choice, the feeding tube, containing a mix of fish flakes (HBH Marine Flake Frenzy, Spanish Fork, UT, USA) and water, was inserted into the aquarium and the food reward was given.

**Figure 2:**
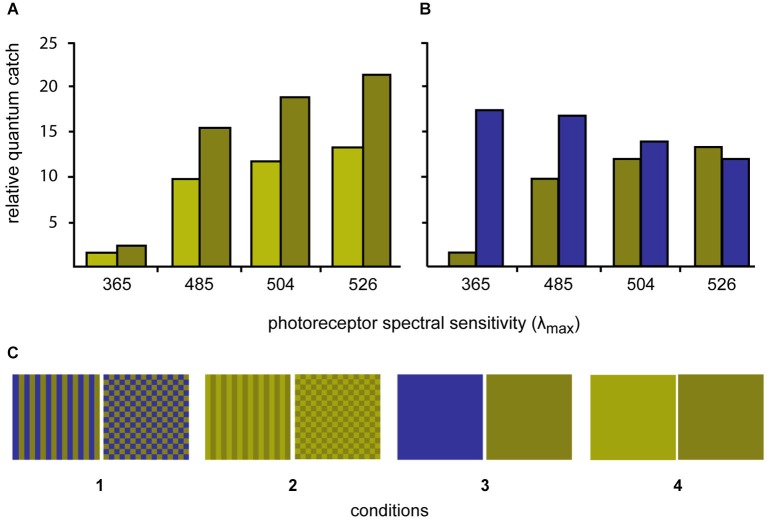
**Quantum catches of the four different photoreceptors of *P. amboinensis* when looking at the three different experimental colors using D65 daylight illumination (top graphs). (A)** Quantum catches for the light green/dark green stimuli are shown while on the right, the quantum catches for the dark green and blue stimuli are compared. **(B)** The dark green and blue colors were selected to minimize contrast to the L-cone (λ_max_ 526 nm) and the light green color was selected to only differ from the dark green color in brightness (but not hue). **(C)** The three colors were combined to form four stimulus conditions (bottom row) with different spatial properties.

**Figure 3:**
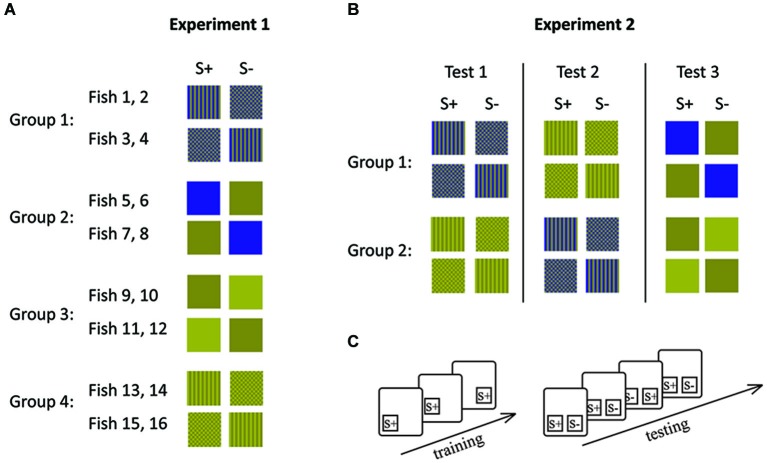
**Stimulus combinations (A, B) and experimental procedures (C) during experiment 1 (A) and experiment 2 (B)**. **(A)** Each group of fish was trained to a different stimulus set and **(B)** each group of fish was retrained following the completion of 10 sessions for a particular stimulus set. Lines indicate retraining events. **(C)** During initial training only S+ was shown in different positions. During testing both stimuli were presented simultaneously. During each session (10 trials), S+ and S− were shown equally often on both sides. S+ indicates the rewarded stimulus and S− the distracter stimulus.

In experiment 1, four fish were trained to each of the four conditions (Figures 2, 3). Within each condition, two fish each were trained to each of the two stimuli (e.g., two fish were trained to stripes and the other two to checkers). This was done in order to control for a possible bias towards a particular stimulus. The second stimulus (distracter) was introduced once the fish had learned to swim to and tap the trained stimulus presented in one of two locations on the board in order to receive a food reward.

Experiment 1 left the possibility open that any difference in performance could be due to some characteristic of the different fish used rather than due to their ability to solve the experimental tasks. In experiment 2, we therefore controlled for this possibility by retraining the fish so that they had to complete both pattern conditions (condition 1 and 2) as well as one condition with solid colors (condition 3 or 4). Each group of fish either started with Condition 1 or Condition 3 (patterns) before completing condition 3 or 4 (solid colors; Figure 3). Fish were randomly allocated to each group.

### Testing procedure

In order to be able to discount a side preference from the selection results the stimuli were presented in random positions counterbalanced across each testing session. The only constraint on the randomization process was that the stimuli never appeared in the same position more than twice in a row. If a fish took more than 2 min to complete the task, the board was removed and the next fish was tested.

Two printed laminated stimuli were attached to a board which was then placed into the aquarium of the fish under investigation (Figures 1, 3). For each trial, the stimuli were randomly chosen from six replicate stimuli thus preventing the fish from using any cues specific to a particular replicate (e.g., slightly different cutting angle of the laminate can cause different reflections).

The stimuli were removed from the aquarium following a correct completion of the task and a food reward or a timeout (2 min). Fish were tested twice a day and made 10 choices in each session. Eight sessions were carried out for each condition in both experiments.

### Analysis

The number of correct choices within each of the eight sessions was determined and, in each case the last four sessions were used for further analysis. This was done to discount the learning phase during the first four sessions. Graphpad Prism was used to carry out the statistical tests. Two-tailed binomial tests were used to determine whether the observed choice frequency of each fish as well as the average response of all fish within each condition was different from chance, i.e., from a 1:1 (distracter: stimulus) selection.

In experiment 1, the hypothesis was tested that the patterns (high frequency stimuli) created with the L-cone matched colors (blue-green patterns) would be harder to discriminate compared to the patterns created with colors that proved L-cone contrast (light green—dark green patterns). We also hypothesized that the solid colors (low frequency stimuli) blue vs. green would be easier to discriminate than the light green/dark green stimuli. Two-tailed *t*-tests were used to analyze the results.

In experiment 2, repeated measures 2-factorial ANOVA was used to test (a) whether training sequence (condition 1 or condition 2 first) influenced the results; and (b) whether there was a significant difference between the fish’s performance in the two pattern conditions.

## Results

### Experiment 1

All fish learned the task of tapping their reward stimulus within 3–4 days of capture so that testing could begin on day 5.

**Figure 4:**
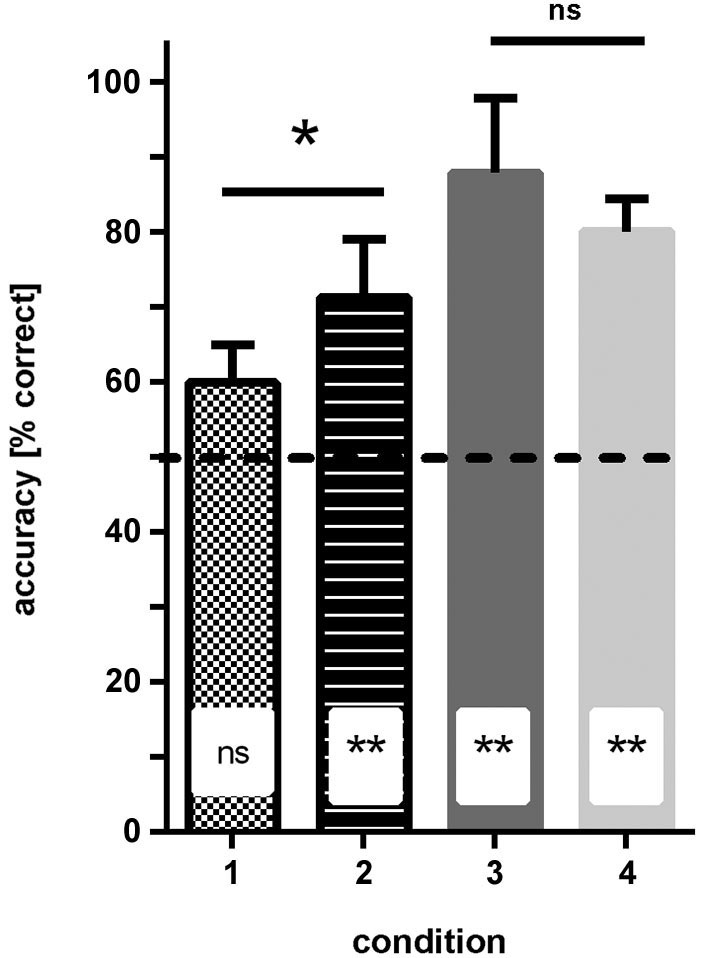
**Results of experiment 1**. The average accuracy (% correct choices) is shown for groups of fish trained to four different conditions (see Figure 1 for details of conditions). No difference in performance was found when the solid color conditions were compared, but performance was significantly worse for fish trained to condition 1 (blue—green patterns) relative to condition 2 (dark/light green patterns; significance levels are given above the bars). Additionally, results are compared to chance level (50% accuracy; insets in bars). ns—not significant, * *p* < 0.05; ** *p* < 0.01.

Condition 1 (patterns: blue/dark-green, stripes vs. checkers): none of the fish reached ≥70% correct choices in two consecutive sessions within the eight testing sessions. In the last four sessions, the fish made on average 59% (sd ±5) correct choices, which was not significantly different from chance (Binomial test: *p* = 0.081; Figure 4).

Condition 2 (patterns: light-green/dark-green, stripes vs. checkers): within 3–4 sessions all fish were able to discriminate the patterns at a level of at least ≥70% correct choices in two consecutive sessions. In the last four sessions, the fish made on average 71.5% (sd ±7.8) correct choices, which was significantly different from chance (binomial test *p* = 0.0088; Figure 4).

Condition 3 (simple colors: blue vs. dark-green): within 3–4 sessions, all fish were able to discriminate the colors and reached a level of at least 70% correct choices over at least two consecutive sessions. In the last four sessions, the fish made on average 88.2% (sd ±10) correct choices, which was significantly different from chance (binomial test: *p* < 0.0001; Figure 4).

Condition 4 (simple colors: dark-green vs. light-green): within 3–4 sessions, all fish were able to discriminate the colors with a frequency of at least 70% correct choices over at least two consecutive sessions. In the last four sessions, the fish made on average 80.3% (sd +/−4.3) correct choices, which was significantly different from chance (binomial test: *p* < 0.0001; Figure 4).

Comparison of different conditions showed that the hypothesis that the green patterns are easier to discriminate than the blue/green patterns is correct (two-tailed *t*-test: *t* = 2.46, df = 6, *p* = 0.048). No significant difference was found between the performance of the fish in conditions 3 and 4 (solid colors, two-tailed *t*-test: *t* = 1.24, df = 6, *p* = 0.26).

### Experiment 2

**Figure 5:**
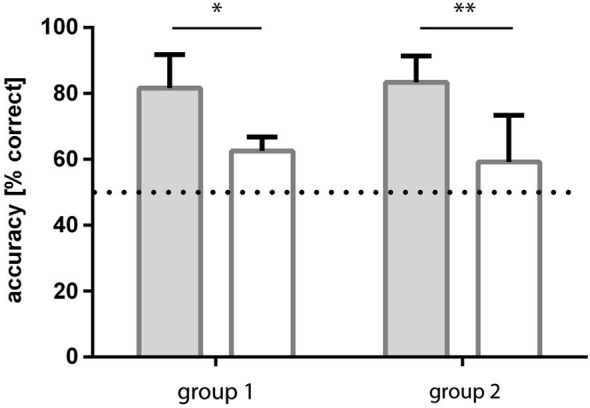
**Results of experiment 2**. Two groups of fish were trained to both pattern conditions, but in a different order. Group 1 fish were trained to light green-dark green patterns first and then retrained to blue-green patterns whereas group 2 experienced the opposite. In both cases, accuracy was significantly higher for dark-light green patterns and results for blue-green patterns were not significantly different from chance.

Group 1: The group of fish initially trained to light/dark green patterns learned to discriminate the checked and striped patterns within 4–5 sessions (all three fish reached a level of ≥70% correct choices). Over the last four sessions, the fish reached an accuracy level of on average 83.3% (sd ±8.0) correct choices (Figure 5).

Following retraining to blue/green patterns, this group of fish was no longer able to discriminate the checked from the striped patterns. They reached a level of 59.2% (sd ±14.1) correct choices over the last four sessions (Figure 5).

Group 2: The fish initially trained to blue/green patterns were not able to discriminate the checked from the striped patterns in this condition. They reached a level of 62.5% (sd ±4.3) correct choices over the last four sessions (Figure 5).

Following retraining to light/dark green patterns they learned to discriminate the checked and striped patterns from the first session on with at least 70% accuracy. Over the last four sessions they reached a level of 81.7% (sd ±10.1) correct choices (Figure 5).

### Comparison of the conditions

The performance of the fish in the two conditions (blue/green and light/dark green) was found to be significantly different (repeated measures ANOVA: F1, 4 = 71.16, *p* = 0.0011), no influence of the training sequence was found (*F*_(1,4)_ = 0.01198, *p* = 0.92) and no interaction existed between the factor training sequence and condition (*F*_(1,4)_ = 0.95, *p* = 0.38). *Post hoc* multiple comparisons showed that performance of the fish was consistent for the two repetitions of each condition (Sidak’s multiple comparison test).

Following the retraining to the second pattern condition the fish were randomly allocated into two groups. One was retrained to blue vs. green simple color, the other to light green vs. dark green. Over the last four sessions, animals allocated to group 1 reached a level of 80% (sd ±10.7) and those allocated to group 2 a level of 65% (sd ±21) correct choices.

## Discussion

Despite the colorful nature of many coral reef fish patterns, limited knowledge exists about the visual processing of color and patterns in fish. In many animals, visual processing of color and luminance is achieved via parallel processing channels. We aimed to test whether there is a spatially selective luminance channel in the coral reef fish, *Pomacentrus amboinensis* using a combination of visual modeling and behavioral experiments based on operant conditioning. We showed for the first time, that, similar to what has been described for terrestrial animals, contrast to L and/or M-cones is required for high spatial frequency pattern discrimination in reef fish.

In the first experiment, we compared the ability of fish to discriminate two colored squares, which differed in either, luminance (light green and dark green), or hue (blue and dark green with near equal L and M-cone quantum catch) or two patterned squares (checkers and gratings) made up of either of the two color combinations. All fish rapidly learned to associate a color or pattern with a food reward within the typical timeframe of 3–4 days post capture, observed in previous studies (Siebeck et al., 2008, 2009). The fish trained to a solid color (blue or dark green) were able to discriminate their rewarded square from another colored square with high accuracy, irrespective of whether the squares differed in hue or brightness.

The fish trained to light green—dark green checked patterns were also able to discriminate their rewarded stimulus from the distractor (light green—dark green gratings), while the fish trained to blue-green patterns (no L and M-cone contrast) were unable to discriminate checkers from gratings. At this point our results could be explained in two possible ways. Either the group of fish trained to this condition were unable to learn or had motivational problems often seen in behavioral experiments (Newport et al., 2014), or L and/or M-cone contrast is indeed required for high spatial frequency pattern discrimination in these fish.

To exclude the possibility of motivational or learning problems, in the second experiment we used a repeated measures design, in which each fish acted as its own control. One group of fish was initially trained to the patterns, which provided luminance contrast only (green—green) and then retrained to the patterns, which did not provide L/M-cone contrast (dark green—dark blue). The other group of fish completed the experiment in the reverse order. Irrespective of the order of the conditions, the fish were only able to discriminate the patterns if contrast was provided for the L and M cones (i.e., the light green—dark green patterns). We can therefore conclude that the loss in discrimination ability in the L/M isoluminant condition was not due to a loss in motivation or learning difficulty, and that contrast to the L and M cones is indeed required for the discrimination of high frequency patterns. Following the pattern discrimination, the fish were retrained a second time to either of the two simple color conditions. Interestingly, the fish allocated to the chromatic contras condition (blue vs. green) solved the task with much higher accuracy compared to those re-trained to the luminance contrast condition (light green vs. dark green). This further demonstrates the spatial selectivity of the luminance channel.

Our findings imply that reef fish also process visual stimuli in separate channels and that not all cones contribute equally to color and luminance vision when processing static patterns. The luminance channel receives input from L and M cones in primates (Livingstone and Hubel, 1988), L cones only in bees (Giurfa et al., 1997) and probably from double cones containing L visual pigments in birds. Due to the electric coupling found in some fish double cones, the long standing hypothesis has been that double cones are the most likely candidates for motion and luminance vision in fish (Boehlert, 1978; Lythgoe, 1979; Cameron and Pugh, 1991; McFarland, 1991). This hypothesis has recently been challenged by a study showing that both double cones can contribute separately to color vision in a reef fish (Pignatelli et al., 2010). Our results show for the first time that L and/or M-cone contrast is essential for pattern discrimination but it is still unclear whether in fish L cones only, or L and M cones contribute to luminance vision. What we can say however is, that, as the double cones in *P. amboinensis* contain the S and L sensitive cones (rather than M/L cones), they do not form the luminance channel as previously proposed and also, that contrast to the S-cone alone is not sufficient for pattern discrimination. While there is previous evidence which supports the existence of a separate channel for large field motion processing in fish (optomotor response is mediated via the L-cones of zebrafish and goldfish; Schaerer and Neumeyer, 1996; Krauss and Neumeyer, 2003), and small field motion processing via M-cones of goldfish (Gehres and Neumeyer, 2007), our study is the first to demonstrate high spatial acuity of the luminance channel in fish.

Overall, it seems that processing visual information in parallel channels is a general feature of visual systems within the animal kingdom, despite many differences in eye design, such as different optics, the morphology and number of photoreceptors with different spectral sensitivities and also perhaps most importantly in brain size and processing power. In primates we know that these parallel channels, i.e., the parvocellular, magnocellular and koniocellular pathways have their origin in the retina and follow all the way through to higher processing centres in the cortex where they feed into the ventral and dorsal streams (Ungerleider and Mishkin, 1982; Yoonessi and Yoonessi, 2011). Whether similar pathways exist in animals with smaller brains and reduced apparent processing power, such as fish and insects is an exciting field for further investigation.

## Conflict of interest statement

The authors declare that the research was conducted in the absence of any commercial or financial relationships that could be construed as a potential conflict of interest.
